# Comprehensive Insight into Cutaneous Application of Hemp

**DOI:** 10.3390/pharmaceutics16060748

**Published:** 2024-05-31

**Authors:** Ana Žugić, Milica Martinović, Vanja Tadić, Miloš Rajković, Gordana Racić, Ivana Nešić, Anamarija Koren

**Affiliations:** 1 Institute for Medicinal Plant Research “Dr. Josif Pancic”, Tadeusa Koscuska 1, 11000 Belgrade, Serbia; azugic@mocbilja.rs (A.Ž.); mrajkovic@mocbilja.rs (M.R.); 2 Faculty of Medicine, University of Nis, Zorana Đinđića Boulevard 81, 18000 Niš, Serbia; milica.martinovic@medfak.ni.ac.rs (M.M.); ivana.nesic@medfak.ni.ac.rs (I.N.); 3 Faculty of Ecological Agriculture, University Educons, Vojvode Putnika 87, 21208 Sremska Kamenica, Serbia; gordana.racic@educons.edu.rs; 4 Institute of Field and Vegetable Crops, Maksima Gorkog 30, 21000 Novi Sad, Serbia

**Keywords:** hemp, natural bio-compounds, circular bioeconomy, topical delivery systems, cosmeceuticals

## Abstract

Known for its natural bio-compounds and therapeutic properties, hemp is being utilized in the development of skin products. These products offer a wide range of applications and benefits in the fields of natural bio-compounds, pharmaceutical technology, topical delivery systems, and cosmeceuticals. This manuscript deals with hemp actives, such as cannabinoids, terpenes, and flavonoids, and their diverse biological properties relative to topical application, including anti-inflammatory, antimicrobial, and antioxidant effects. Also, the paper reviews strategies to overcome poor penetration of hemp actives, as well as the integration of hemp actives in cosmeceuticals that provide natural and sustainable alternatives to traditional skincare products offering a range of benefits, including anti-aging, moisturizing, and soothing properties. The review aims to provide a comprehensive understanding of the development and manufacturing processes of skin products containing hemp actives. By delving into the science behind hemp-based products, the paper provides valuable insights into the potential of hemp as a versatile ingredient in the pharmaceutical and cosmetic industries. The utilization of hemp in these innovative products not only offers therapeutic benefits but also promotes natural and sustainable approaches to skincare.

## 1. Introduction

**Figure 1:**
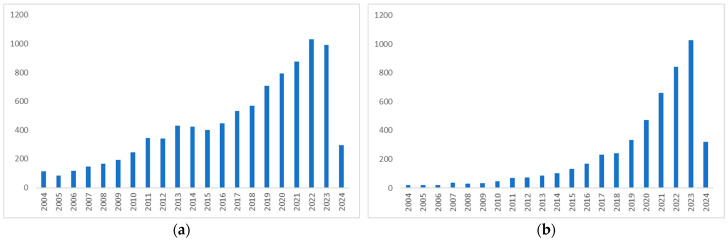
Expansion of scientific research on hemp found on Scopus in the last 20 years (2004–2024): (**a**) 9255 papers were found when the search was restricted to the term “hemp” as the keyword, (**b**) 4961 papers were found when the search was restricted to the term “hemp” and “skin” in all fields.

For centuries, hemp has found application across various industries, including textiles, food, feed, and construction. However, it is the realm of health and wellness that has recently sparked increased curiosity and scientific exploration into hemp’s potential benefits for human well-being. Particularly noteworthy is the growing interest in the therapeutic and cosmetic utilization of hemp and its derivatives into products for cutaneous application (Figure 1), while they are believed to offer a spectrum of advantages possessing a multitude of beneficial properties, including antibiotic, antimicrobial, anti-inflammatory, and antioxidant activities [1]. While the evidence base for the diverse benefits of hemp actives is still nascent, the potential for further research and the development of targeted skin products is vast. This paper offers a comprehensive insight into the utilization of hemp with reference to hemp activities and scientific data regarding their potential for skin application, while at the same time offering emphasis on legal issues and quality assurance of these products, as well as challenges in their development within the context of the circular bioeconomy.

For the purpose of this review, a comprehensive electronic survey was conducted using several search tools, including Scopus, Web of Science, and Medline, using the term “hemp” as the keyword and “skin” in all fields, with the search period spanning from 2004 to 2024. The guidelines and documents from the relevant organizations, such as the Food and Drug Administration (FDA) and the European Medicines Agency (EMA), were also consulted when appropriate.

## 2. Natural Bioactive Compounds (NBCs) of Hemp

**Figure 2:**
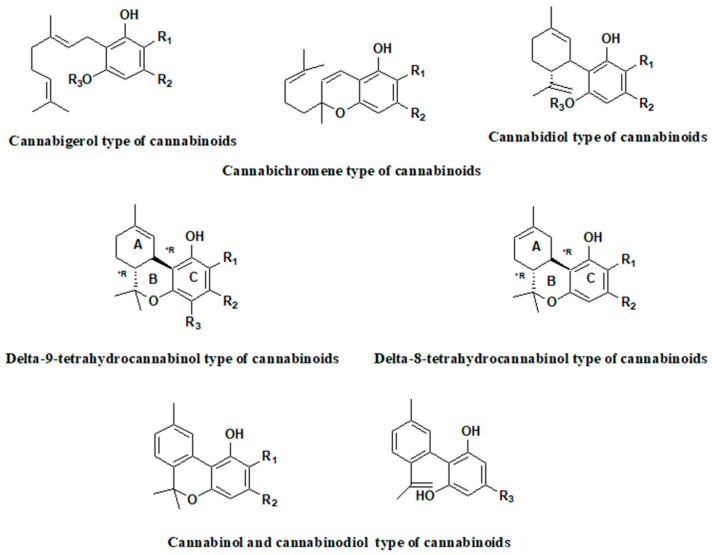
The main cannabinoids present in the hemp extracts.

**Figure 3:**
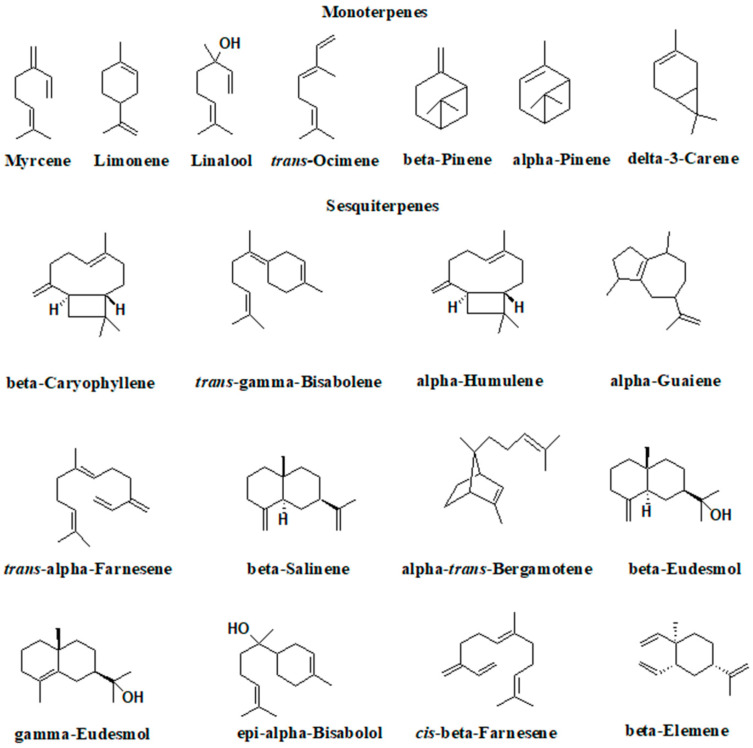
Terpenoids present in essential oils are obtained from the hemp aerial part.

The term “hemp” (*Cannabis sativa* L., *Cannabaceae*) refers primarily to a plant grown as an agricultural crop characterized by a low content of psychoactive Δ^9^-tetrahydrocannabinol (THC, less than 0.3%). Numerous studies on the medical applications of hemp have been initiated, causing an increase in scientific knowledge regarding its composition and health benefits. Much attention has been focused on non-psychoactive cannabinoids (cannabidiol, CBD, cannabinol, CBN, and cannabichromene, CBCr) (Figure 2), which represent the most promising candidates for clinical utilization due to their lack of cognitive and psychoactive actions but with significant anti-inflammatory and neuroprotective properties. The therapeutic effects, manifested in the modulation of inflammatory processes, can be explained by cannabinoids’ multiple mechanisms of action. To date, two cannabinoid receptors through which the activity of some cannabinoids might be achieved are known as CB1 (mainly positioned in the CNS) and CB2 (principally expressed in immune cells but also found in various other cell types, including chondrocytes, osteocytes, and fibroblasts, meaning that it can be considered the peripheral cannabinoid receptor). While CB1 exerts psychoactive activity, activation of CB2 has, as a consequence, immunomodulatory and anti-inflammatory effects. Hence, controlling the degree of inflammation by selectively acting on CB2 receptors might be responsible for the desired anti-inflammatory activity. In addition, cannabinoids’ anti-inflammatory properties might be exerted at the cellular level due to their antioxidant capacity as well as their ability to affect cell wall properties. The most promising anti-inflammatory substance among cannabinoids is CBD. Namely, CBD acts as an inverse agonist of CB2 and might represent the molecule of choice for topical treatments. Also, it has been shown that CBD is a potential COX-2 and LOX-5 inhibitor targeting the arachidonic acid pathway related to inflammation response. Besides the cannabinoids (Table 1), other components, such as the monoterpenoids myrcene, limonene, and pinene, and the sesquiterpenoid β-caryophyllene (Figure 3), can also mediate the pharmacological effects of hemp [2,3,4].

**Figure 4:**
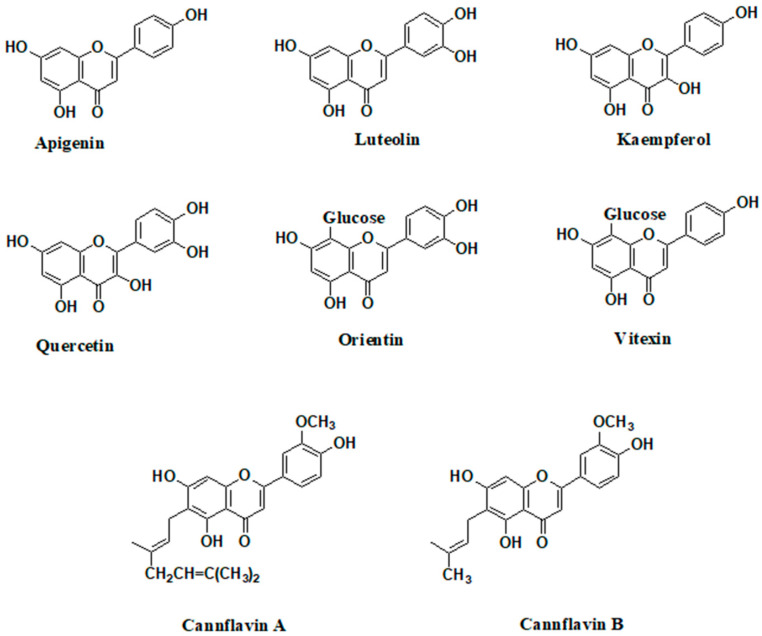
C- and O-forming flavonoid aglycones and C-glycosides are present in hemp extracts.

It has recently been shown that cannabinoids appear to be opportune active molecules for the local treatment of anti-inflammatory skin disorders, arthritis, and peripheral neuropathic pain. Myrcene, the most abundant monoterpene in hemp essential oil, has analgesic, anti-inflammatory, antibiotic, and antimutagenic properties. β-Caryophyllene, the most common sesquiterpene, exhibits anti-inflammatory, cytoprotective (gastric mucosa), and antimalarial activity. Apigenin, a flavonoid found in hemp, exerts a wide range of biological effects, including many properties shared by terpenoids and cannabinoids. Apigenin inhibits the production of tumor necrosis factor-alpha (TNF-α), a cytokine primarily expressed by monocytes and macrophages that induces and maintains inflammation. Quercetin, a flavonol, is a potent antioxidant, considered even more potent than ascorbic acid, α-tocopherol, and BHT. Combinations of quercetin and other antioxidants work synergistically. Flavonoids (Figure 4) block free radical formation at several steps: by scavenging superoxide anions (in both enzymatic and non-enzymatic systems), by quenching intermediate peroxyl and alkoxyl radicals, and by chelating iron ions, which catalyze many Fenton reactions leading to free radical formation. Cannflavin A is one of a pair of prenylated flavones unique to hemp. This compound is a potent inhibitor of prostaglandin E2 in human rheumatoid synovial cells. Cannflavin A inhibits cyclooxygenase (COX) enzymes and lipoxygenase (LO) enzymes [2,3,4].

The essential oil from hemp inflorescences is responsible for the flavor of the hemp. It is rich in sesquiterpenes, especially β-caryophyllene and α-humulene, but also in monoterpene hydrocarbons (β-myrcene) and oxygenated sesquiterpenes. The phytocannabinoids are also present as the second major chemical class, following terpenes [5].

**Figure 5:**
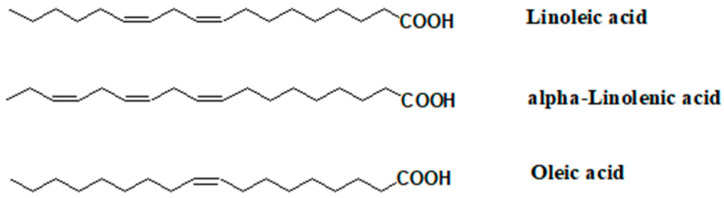
Unsaturated fatty acids from hemp seed oil.

On the other hand, the hemp roots are rich in friedeline, epifriedeline, carvone, dihydrocarvone, and β-sitosterol [6]. Similarly, in hemp seeds, unlike leaves, flowers, and essential oils, cannabinoids and terpenes are present in trace amounts. Hemp seed oil, rich in ω-3, ω-6, and ω-9 acids (Figure 5), is obtained in most cases by mechanical cold pressing of the seeds, but also by solvent extraction, microwave or ultrasound-assisted extraction, or by supercritical extraction using carbon dioxide [7]. Major components are polyunsaturated fatty acids (PUFAs) such as γ-linolenic acid (GLA); essential fatty acids (EFAs), especially linoleic acid and α-linolenic acid; and monosaturated fatty acids—oleic acid [8]. This oil is famous for its favorable ω-6 to ω-3 EFA ratio (2–3:1). Hemp seed oil contains carotenoids (β-carotene, lutein, and zeaxanthin), tocopherols (α-, β-, γ-, and δ-tocopherol), phytosterols (campesterol and β-sitosterol), chlorophyll, and phenols (flavonoids and polyphenols) as well. Besides oil, hemp seeds are also composed of carbohydrates, dietary fibers, and minerals [3,8].

## 3. Overview of Research Studies on the Effects of Hemp NBCs Relative to Potential Usage in Products for Cutaneous Applications

### 3.1. Skin Endocannabinoid System

The assumption that phytocannabinoids can affect skin health and contribute to skincare stems from the knowledge that the presence of the endocannabinoid system (ECS) in the skin is of enormous importance for skin metabolism. ECS regulates cell growth and differentiation as well as immune and inflammatory responses (Table 2). The endocannabinoids produced in the skin, *N*-arachidonoyletahnolamine (AEA) and 2-arachidonoylglycerol (2-AG), are binding to the main endocannabinoid receptors CB1 and CB2, as well as other receptors—TRPV1 belonging to Transient Receptor Potential (TRP) channels and PPARγ and PPARα from the Proliferator-Activated Receptors (PPAR) family [3,9].

### 3.2. Biological Activities

Antioxidant activity. Many mechanisms are proposed for explaining phytocannabinoids’ antioxidant activity. Phyitocannabinoids (CBD, CBN, CBG, CBC, and THC) can express redox behavior and act as lipophilic electron donors [10]. CBD can reduce ROS formation in keratinocytes via the NRF2-hemoxigenase-1 signaling pathway [11]. It was also shown that CBD can protect against lipid peroxidation [12,13]. In addition, CBD can influence the activation of antioxidant enzymes in the skin—superoxide dismutase (SOD) and glutathione peroxidase (GPx) [14].

Anti-inflammatory activity. The basis of CBD application in products intended for treating dermatological conditions such as acne, atopic dermatitis, or psoriasis is its ability to modulate the skin’s inflammatory response [15]. CBD inhibits nuclear factor kappa B (NF-κB) and its inflammatory signaling pathway via stimulation of the kinase IKK (IκB kinase) [12].

Hair growth modulation. The relationship between phytocannabinoids and hair growth is rather complex. While some cannabinoids can cause hair growth, others can cause hair loss [16]. According to an ex vivo study conducted on the microdissected organ-cultured human scalp and primary human outer root sheath keratinocytes isolated from plucked hair shafts, CBD can act as a hair growth modulator since it is hypothesized that it may bind to different receptors depending on the concentration. While submicromolar concentrations of CBD-activated adenosine receptors reduced intrafollicular production of cytokines that stimulated the catagen phase of hair loss, micromolar (≥10 μM) concentrations of CBD-activated TRPV4 resulted in the inhibition of hair growth [17]. In a web-based survey conducted in 2021, completed by 1087 participants, the results indicated that many patients with alopecia areata use cannabis or derivatives (CBD, CBD oil, CBD lotions, THC, etc.), but only to relieve psychosocial symptoms. However, they have not noticed hair regrowth [18].

Wound healing. The wound healing ability of CBD and THC was demonstrated in vitro on stress-induced premature senescent fibroblasts using a scratch assay. Those phytocannabinoids showed better effects compared to metformin, rapamycin, and triacetylresveratrol [19].

Anti-age activity. A number of cannabis-infused beauty products have recently hit the market, accompanied by some unverified claims regarding their skin benefits. Existing scientific data, although limited, indicates that hemp may possess beneficial properties suitable for incorporation into anti-aging or rejuvenation products. Nevertheless, the full extent of these potential benefits remains uncertain. In an in vitro study on CCD-1064Sk fibroblasts, CBD and THC caused a reduction in skin aging (according to beta-galactosidase activity) and stimulation of hair growth [19]. CBD also demonstrated cytoprotective activity against UV-induced changes in fibroblasts and prevented collagen degradation via activation of the PI3K/Akt pathway [12]. Overall, phytocannabinoids can protect against UVA and UVB radiation [10]. The moisturizing effect of CBD established through increased expression of aquaporin-3 also contributes to its anti-aging effect [20]. 

Melanin synthesis and anti-tyrosinase activity. Melanocytes are skin cells that produce endocannabinoids, which, at high concentrations, stimulate melanocyte apoptosis, while at low concentrations, they activate tyrosinase and melanogenesis. Therefore, the logical assumption would be that phytocannabinoids could also play an important role in melanogenesis [21]. However, the results obtained are contradictory. Methanol and hemp flower extracts inhibit tyrosinase activity and melanin synthesis [22]. The same was observed for minor cannabinoids—CBN, CBC, and CBG [21]. On the other hand, CBD and THC stimulate in vitro tyrosinase activity and melanin synthesis in human epidermal melanocytes [23,24]. 

Antibacterial activity. Sixty years ago, it was found that CBD can act against Gram-positive bacteria [25]. Wassmann et al. confirmed the synergistic activity of CBD with bacitracin against Gram-positive bacteria, which can be helpful in the treatment of cutaneous infections, while CBD would exhibit dual action—antimicrobial and anti-inflammatory activity [26]. Cannabidiolic acid (CBDA) has also been linked to antibacterial activity [27]. In the study of Novak in 2001, hemp essential oil showed modest antimicrobial activity [28], while in other studies, an antibacterial effect was detected against *Pseudomonas aeruginosa*, *Bacillus subtilis*, *Escherichia coli*, *Micrococcus luteus*, and *Staphylococcus aureus* susp. *aureus* [29,30]. Moreover, refined oil showed better bioactivity compared to unrefined oil, which might be a consequence of the long-chain unsaturated fatty acid content (oleic, linoleic, and linolenic) [30]. In addition, hemp seed oil inhibited the growth of the yeast [31]. However, it was not active against *Candida albicans* or *Aspergillus niger* [29].

The summary of the biological activities of hemp derivatives is presented in Table 3.

### 3.3. Potential Indications for Application of Hemp-Based Products

Psoriasis. Psoriasis is a chronic skin disease associated with strong pro-inflammatory activity and oxidative stress, leading to dysregulated keratinocyte differentiation. Cannabinoids (THC, CBD, CBN, and CBG) can inhibit keratinocyte proliferation via PPARγ receptors and, therefore, can be beneficial in the treatment of psoriasis [33]. The functioning of ECS is disturbed in psoriasis, and CBD can increase the expression of CB1 and CB2 receptors. Jarocka-Karpowicz et al. have found that UV-irradiated cells from healthy and psoriatic patients act differently after treatment with CBD. While oxidative stress was decreased in healthy cells, the situation was the opposite in psoriatic cells [13]. Therefore, more studies need to be conducted to understand the potential role of cannabinoids and ECS in the pathogenesis and treatment of psoriasis.

Atopic dermatitis. This chronic skin disease affecting a large number of children and adults is linked to epidermal barrier dysfunction coupled with a prolonged Th2-type inflammatory state [34]. Hemp seed oil, due to its specific essential fatty acid content with a preferred omega-6 to omega-3 ratio (1:3), can significantly contribute to skin health and renew skin barrier function. This is why hemp seed oil, which mostly contains phytocannabinoids only in traces, can alleviate symptoms of atopic dermatitis [35]. However, the symptoms of atopic dermatitis can also be improved by CB receptor agonists and their interaction with skin ECs [36]. 

Acne. Since CB2 receptors were detected in human SZ95 sebocytes, the potential of CBD application in acne vulgaris treatment has been examined in a couple of studies [37]. In vitro studies on cultured human sebocytes indicated that CBD has lipostatic activity. Moreover, anti-inflammatory activity can be explained by binding to A2a receptors, activating the production of cAMP, and, consequently, inhibiting the NF-κB pathway. Also, CBD can suppress sebocyte proliferation by activating transient receptor potential vanilloid-4 (TRPV4) ion channels and by downregulating nuclear receptor interacting protein-1 (NRIP1) [32]. The vitality of sebocytes can be changed by smaller doses of phytocannabinoids (up to 10 μm), while higher doses (≥50 μm) can lead to sebocyte apoptosis [38]. These findings were confirmed in an in vivo study where daily application of 1% hemp seed oil cream (0.2% CBD) led to improvement of acne lesions and reduction in sebum level while not causing serious adverse effects [39].

Hyperhidrosis. It has been shown that the consumption of dronabinol drops for a month and later inhalation of medical cannabis buds (8% THC and 8% CBD) may lead to a decrease in transpiration and sweat volume, according to a case report of a 28-year-old male [40].

## 4. Strategies for the Enhanced Topical Delivery of Hemp NBCs

One of the most favorable routes of product application by patients is topical drug delivery [41] since it is based on the direct application of the formulation to the body’s exterior areas; this method is non-invasive, painless, convenient, and easy. However, the skin, as the largest human organ and the outmost layer, represents a complex barrier whose role is to protect from the external environment, thus setting the challenge for researchers to develop suitable formulations that could ensure and enable the delivery of active principles to the intended site [42,43]. The major barrier is the *stratum corneum*, which represents the physical barrier for the passage of molecules with a molar mass over 500 Da because of its unique brick-and-mortar structure of cells planted in a lipid matrix. Beneath the stratum corneum and epidermis, there is a thick dermis containing blood vessels that are the gateway to the systemic circulation [44].

Due to its molecular structure, one of the most studied hemp ingredients—CBD—is characterized by poor hydrosolubility and a high logP (lipid–water partition coefficient) value, which is why its permeation through viable epidermis is limited [45]. THC and CBN, compared to CBD, are even more lipophilic. One of the reasons is that they have one less hydroxyl group in their chemical structure [46]. 

Chemical permeation enhancers, intended for temporarily reducing its barrier function without destroying the cells, can be used for improving skin permeability, as well as some methods like microdermabrasion, microneedles, and thermal ablation [47]. For instance, oleic acid as a penetration enhancer helped the delivery of THC across rat and human skin, while decylmethylsulfoxide, in combination with oleic acid, increased the delivery of THC on hairless murine skin [48,49,50]. In an in vitro study on human skin samples, ethanol increased the permeation of THC and CBD nearly three-fold [51]. 

Numerous investigations are focusing on developing appropriate carrier systems and formulations for improving cannabinoid bioavailability and enabling its therapeutic effect. Defining a suitable vehicle for new active substances always poses a challenge since many elements like solubility, thermodynamic activity of active substances, or characteristics of the vehicle need to be considered [52]. In this respect, Casiraghi et al. have compared the permeation of CBD in four solutions and two semisolid formulations in an in vitro test using Franz diffusion cells mounted on human skin [52]. Interestingly, it was found that a hydrophilic gel, mostly consisting of propylene glycol, offered better performance in terms of topical CBD delivery compared to lipophilic ointment. On the other hand, a comparison of the hydrophilic gel to the corresponding propylene glycol solution in the same CBD concentration in this study revealed better performance of the gel regarding both permeation rate and skin retention. Nevertheless, it has been shown that CBD was quite unstable in a solution for a long period of time [52]. This is why there are a variety of ideas and attempts to enhance both the stability and delivery of CBD, starting with basic galenic preparations and leading to complicated and innovative nanotechnology approaches [44]. Currently, CBD is being used as an oil dispersion in hemp, sesame, soybean, coconut, and olive oil [53]. However, oils are prone to oxidation and oxidative degradation, which is why they are not the most desirable carriers [54]. Alternatives are emulsion systems, both water-in-oil and oil-in-water types, which not only offer better stability and bioavailability but are also better accepted by patients due to their applicative characteristics [55]. Based on the results of the DCS-TGA analysis, CBD is compatible with commonly used excipients for emulsion system formulation, such as non-ionic surfactants polysorbate 20, polysorbate 80, sorbitan monooleate 80, and sorbitan monooleate 85, as well as natural oils such as sesame, soybean, olive, and safflower oils, while it is not compatible with caprylic/capric triglycerides [56].

Not only active substances isolated from hemp but also hemp extracts and derivatives have been incorporated in emulsions. Dried hemp seed methanol/water extract in a concentration of 3% was incorporated into water-in-oil cream stabilized with 2% Abil EM 90. The cream contained 14% paraffin oil as an oil phase constituent [57]. Hemp seed oil in both refined and unrefined form was incorporated as a dispersed phase in stable oil-in-water emulsions stabilized with non-ionic emulsifiers’ combination of sorbitan monooleate 85 and polysorbate 85, while the greatest stability was achieved with a 10% concentration of surfactants at HLB 9 [30]. The influence of processing parameters (temperature, storage, and light exposure) on the process of oxidation of hemp oil as a dispersed phase in emulsion with soy lecithin as an emulsifier was investigated. The results have shown that the hemp oil in the aqueous environment of the emulsion can be susceptible to oxidation catalyzed by light and temperature and, therefore, demands the presence of antioxidants in the formulation [58].

**Figure 6:**
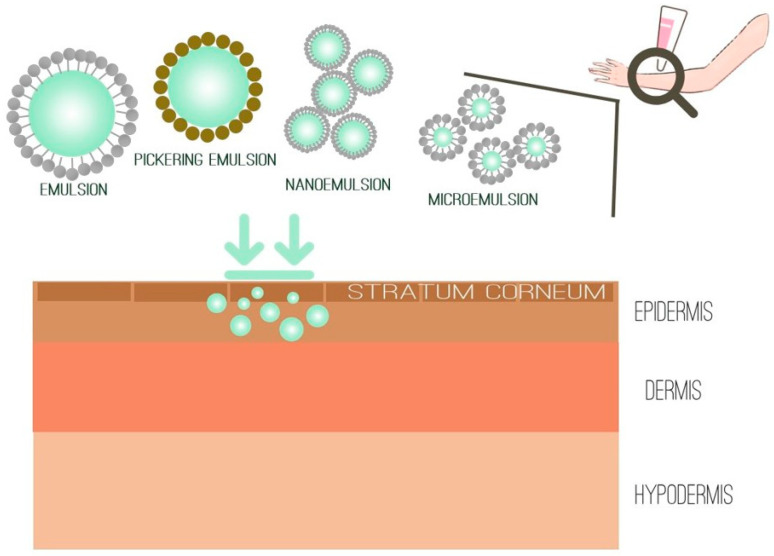
Emulsion-type vehicles for topical drug delivery.

The most abundant type of dermocosmetic product on the market is emulsions (Figure 6). However, various types of nano-delivery systems (nanoemulsions, microemulsions, liposomes, vesicles, nanostructured lipid carriers, solid lipid nanoparticles, etc.) have been developed with the aim of improving efficacy and delivery of actives to the skin [59].

Lipogel, composed of water, phenoxyethanol, caprylyl glycol, decylene glycol, carbomer, and glycerin, containing hemp seed oil and CBD, among other active substances, was developed with the purpose of topical analgesic activity. This emulgel, as an emulsion whose water phase was gelled with carbomer as a gelling agent, has been suggested as an alternative to local nonsteroidal anti-inflammatory drugs used in the treatment of joint pain [60].

In order to overcome the problem of the lack of skin permeation of hemp oil-based products, nanoemulsions with hemp seed oil and hemp seed oil microparticles were developed [61]. The problem with formulating nanoemulsions is the fact that not only the active substance (hemp seed oil in this case) but also the emulsifier itself is being decreased to nanosize, which opens the possibility for deeper penetration and potential skin irritation [62]. Lubart et al. were able to develop micronized hemp without the use of surfactants and to incorporate it in the cream, which showed good antioxidant and anti-inflammatory activity [61]. CBD was also incorporated in stable oil-in-water nanoemulsions and nanoemulsion-filled chitosan hydrogels [63].

Microemulsions as dermatologically stable, transparent, monophase colloidal dispersions are very useful in topical drug delivery. Vanti et al. [64] formulated and characterized a new CBD-based microemulgel that could potentially be used for the treatment of various dermatological disorders. The microemulgel was formulated by gelling the water phase of the microemulsion with a suitable gelling agent (Sepigel 305, carbomer, and Xanthan gum). The obtained microemulgel showed better texture characteristics compared to the mother microemulsion from which it was formed, optimal physicochemical properties (stability, pH, viscosity, and uniformity of CBD content), and a desired release profile since good retention of CBD in the skin was observed without systemic resorption [64].

Pickering emulsions are also emulsion-stabilized without the use of surfactants. Chitosan and collagen peptides were used as biocompatible and biodegradable vehicles with high encapsulation efficiency for the formulation of Pickering emulsions with CBD. This formulation enabled high deposition of CBD in the epidermis [65].

For the purpose of wound dressing, porous bacterial levan-based sponges enriched with cannabis oil rich in CBD were fabricated and characterized. The sponges showed the desired attributes expected from the wound dressing, such as biocompatibility, porous structure, and adequate water vapor transmission rate. They were non-toxic and non-allergenic, easy to apply, and painless to remove. In addition, they inhibited microbial growth, showed good antioxidant and anti-inflammatory properties, and were active for 24 h [66].

So far, there are no universal standards regarding products for cutaneous application with cannabinoids and hemp derivatives; thus, the safety and efficacy of each preparation have to be evaluated individually. Standardizing the concentration of CBD in products for cutaneous application cannot be a good indicator of CBD bioavailability due to the lack of a complete understanding of transcutaneous absorption [67]. In addition, by varying the vehicles used in the formulation of CBD, skin permeation could be modulated. An ointment and hydrophilic gel with hydroxyethylcellulose were tested with four different solvent systems (liquid paraffin, propylene glycol, virgin olive oil, and PEG 400) in terms of drug permeation and retention in the epidermis via Franz diffusion cell. Ointment with PEG 400 showed the highest tendency for retention in the epidermis, while gel with propylene glycol enabled the highest permeation of the drug through the lower epidermal layers [52].

## 5. Case Studies of Cutaneous Application of Hemp-Based Products

There is a scarcity of data regarding the impact of applying hemp actives on humans, primarily because there are only a few clinical studies available. Only about 1000 people have so far participated in studies examining the effect of hemp-based preparations on the skin with various dermatological problems. Having in mind that these people suffered from a variety of different dermatological conditions (atopic dermatitis, psoriasis, acne, etc.) and were not exposed to hemp-based products only by topical application but also by oral administration and inhalation, it can be concluded that the amount of clinical evidence is still limited. Further randomized, controlled studies need to be conducted [68].

Many dermatological conclusions were drawn from studies in which the participants took hemp-based products orally. In 2005, a study was conducted where 10 participants with atopic dermatitis took 30 mL of hempseed oil per day for 8 weeks. The results showed that the symptoms of atopic dermatitis were mitigated, and plasma fatty acid levels were increased [69]. 

When it comes to topical application, in a single-blinded, randomized controlled trial, the cream containing 3% cannabis seed extract was applied for 12 weeks twice a day on the cheeks of 11 healthy participants. The research proved that the extract was safe, and its application led to a decrease in cheek sebum production as well as a lowering of the erythema index [57]. In a clinical study by Maghfour et al. [70], the effects of CBD were assessed on 14 patients with atopic dermatitis. During the period of two weeks, they applied a 1% CBD-infused gel with dimethicone, polysilicone-11, and hemp oil as a source of CBD (without THC). The gel led to a significant reduction in eczema severity and intensity of pruritus (29%), which was confirmed by the Eczema Area and Severity Index (EASI), Visual Analogue Scale Pruritus (VAS), and 5-D Pruritus Scales that were used for the evaluation. Out of 14 participants, five of them noticed side effects during application, which manifested as stinging and discomfort [70].

The Psoriasis Area Severity Index (PASI) is used for measuring the severity of psoriasis based on grading erythema, desquamation, and induration. In a randomized, double-blind clinical trial, 51 patients were instructed to apply a 2.5% CBD ointment for 12 weeks. The mean PASI score was lowered significantly by 0.197 [71].

A total of 20 adult patients, out of whom 5 had psoriasis, 5 had atopic dermatitis, and 10 had scars, were involved in the retrospective study, where they were instructed to apply a CBD-enriched ointment (without THC) twice a day to skin lesions for a period of three months. Biophysical skin parameters (TEWL, hydration, and elasticity) significantly improved, while no side effects were detected during the study [72].

Anti-inflammatory shampoo containing CBD and ketoconazole used for two weeks caused a significant decrease in inflammation and erythema manifestation, relief of itching and burning sensation [73].

In a double-blinded, randomized controlled trial, the safety of hemp seed extract was examined, as was its efficacy in the treatment of acne vulgaris. The cream containing 1% hempseed oil and stabilized with non-ionic mixed emulsifier Lipomulse Luxe (INCI: Cetearyl Alcohol (and) Glyceryl Stearate (and) PEG-40 Stearate (and) Ceteareth-20) was used twice a day for 12 weeks by 20 patients with mild to moderate acne vulgaris, according to the Global Acne Grading System. The results have shown that both inflammatory and non-inflammatory acne lesions were improved, while sebum levels were significantly decreased [39].

Epidermolysis bullosa (EB) comprises a cluster of congenital blistering dermatoses that impact the skin and mucosa (bullae, blisters, and scars) due to inherited deficiencies in anchoring proteins responsible for the connection between the epidermis and dermis. There is a case report that self-initiated use of CBD oil by three patients with this dermatologic condition led to improvement of clinical presentation and symptom alleviation [74].

Even though the reports about the influence of phytocannabinoids on hair growth are indecisive, there is a case study reporting that 35 participants with androgenic alopecia used daily 3–4 mg of CBD via topical hemp oil formulation for 6 months, which led to an increase in hair without adverse effects [75]. The same author conducted a new case study with hemp extract rich in tetrahydrocannabivarin (THCV), cannabidivarin (CBDV), CBD, menthol, and peppermint oil on 31 subjects, which led to even better results that were superior to finasteride and 5% minoxidil application [76].

The anti-age effects of retinol are well known, as are the side effects associated with it (redness, drying, and peeling). Few et al. formulated a cream that contained both 0.2% retinol and 300 mg of CBD, which was later applied once daily by ten healthy adults. The idea behind the formulation was that CBD could alleviate retinol side effects. Researchers also suggest that CBD can even express synergistic anti-age activity with retinol [77]. Another combination was tested for anti-age activity. CBD with eicosapentaenoic acid (EPA) was incorporated into cream, which was first tested in vitro and ex vivo and later applied by 34 female participants twice a day for 56 days, leading to an increase in skin hydration and elasticity, along with a reduction in wrinkles and red spots. A combination of CBD and EPA was able to reduce the inflammatory response associated with UV exposure [78].

## 6. Regulatory Considerations for Manufacturing Hemp Products for Cutaneous Application

In the past decade, a notable growth in the hemp market could be observed, particularly for cannabinoid-based skin products. This is a consequence of the adoption of three legal acts in the USA—the Farm Act from 2014, the Farm Bill from 2018, and the Hemp Advancement Act from 2022, which led to the removal of hemp and its derivatives from the US-controlled substances list [79,80,81]. In January 2019, the World Health Organization (WHO), through its Expert Committee on Drug Dependence, recommended alterations regarding cannabis preparations containing CBD without THC. Namely, the proposal emphasized that CBD preparations with less than 0.2% THC should not be subject to international drug control, given CBD’s non-intoxicating nature, general tolerability, and absence of evidence indicating problematic use or associated public health issues, as well as the therapeutic benefits of treating childhood epilepsy [82].

Nowadays, it is estimated that the global market for cosmetics containing CBD will be worth USD 3.5 billion by 2026, with a CARG of almost 25% [83], which confirms that the novel market trend is the proliferation of CBD cosmetics. Nevertheless, this suggests that there is a need for consistent and clearly defined global regulations and, subsequently, national laws governing the incorporation of hemp into skin products, including both medications and cosmetics, a situation that is presently lacking.

The cosmetics regulation lists hemp extracts and oils as non-prohibited plant extracts. However, their THC content and, sometimes, THC:CBD ratio are strictly limited. While the concentration of THC in products for cutaneous application is limited to 0.1–0.3%, depending on the nation and regulatory level, there is no defined acceptable CBD level [84].

In the USA, the use of CBD in cosmetics is allowed and considered safe forasmuch as the concentration of THC is beneath the defined limit of 0.3%. In 2022, a proposal was made for the Hemp Act, which would permit the cultivation of hemp containing up to 1% THC on a dry-weight basis during harvest. However, the maximum concentration of THC in hemp products has to remain below 0.3%. Additionally, in the USA, each state has its own regulation considering the maximum THC concentration allowed in the product and THC:CBD ratio, as well as product testing requirements [85]. In Canada, even though the use of recreational marijuana is legal, the THC content is limited in cosmetic products to 0.3%. 

In Europe, according to Regulation (EU) 2021/2115, the highest allowable THC content in hemp plants is 0.3%. When it comes to hemp usage in cosmetics, European Commission regulation No. 1223/2009 lists approved and forbidden substances in cosmetics within the European Union. Within Annex II, where over 1600 substances are marked as prohibited, there is entry No. 306 named “Narcotics, natural and synthetic”, referring to substances listed in the 1961 Convention on narcotic drugs, where cannabis flowering and fruiting tops and its derivatives are also included. This means that extracts from other parts of the hemp plant and synthetically produced CBD are allowed in cosmetics. In the CosIng database of substances and ingredients that could be used for cosmetics manufacturing, CBD is listed with the INCI names Cannabidiol—synthetically produced and Cannabidiol—derived from the extract, tincture, or resin of cannabis within the same CAS number 13956-29-1 as an ingredient with the following cosmetic functions: antioxidant, anti-sebum, skin conditioning, and skin protection. Cannabidiol trisiloxane is also listed as an ingredient that has a film-forming and dispersing non-surfactant function. *Cannabis sativa* seed oil (CAS number 89958-21-4) and hydrogenated hemp seed oil, as the end products of the hydrogenation of *Cannabis sativa* seed oil, are identified as ingredients with skin conditioning and emollient functions [86]. As in the US, regulations in Europe vary from country to country. In Portugal, substances extracted from hemp seeds with a THC content of less than 0.2% are allowed, while in France, there is an intention to ban the use of CBD in cosmetics due to potential reproductive toxicity. Malta has become the first European country to legalize marijuana [87]. The European Commission published on the 1 June 2023 a call for data on ingredients used in cosmetic products that are active until 30 September 2024, and this call is addressed to researchers, cosmetics manufacturers, and CBD producers to provide information about the safety of pure CBD and extracts containing CBD and other cannabinoids like THC as contaminants [88]. 

In Asia, CBD is not on the list of preferred cosmetic ingredients since it is prohibited in China, Indonesia, Vietnam, Singapore, and the Phillippines. In Japan, CBD can be used as a cosmetic ingredient, but it is necessary that no THC be detected [82].

In Australia, CBD cosmetics (containing less than 50 ppm of THC) can only be purchased by prescription [82]. 

## 7. Quality Control and Testing in the Development of Hemp-Based Products for Cutaneous Application

Due to the intricate nature of *C. sativa* and the diversity of the composition of chemical substances in different parts of the plant (phytocannabinoids, terpenes, volatile compounds, sugars, and fats), efficiently and consistently extracting phytocannabinoids from plant material and cannabis-based products while obtaining precise and dependable potency data poses a significant challenge. Furthermore, the lack of standardized preparation procedures for hemp-based infusions adds another layer of complexity to the situation. Many methods could be used for sample preparation, such as maceration, solid–liquid extraction, liquid–liquid extraction, pressurized liquid extraction, headspace solid-phase microextraction, supercritical fluid extraction, focused ultrasound extraction, ultrasonic-assisted extraction, solid phase extraction, microwave-assisted hydrodistillation, cloud point extraction, and centrifugal partition chromatography, while instrumental analysis is usually conducted using GC- and LS-based methods due to their accuracy, sensitivity, and selectivity. In addition, TLC and HPLC methods are also being used, as well as capillary electrophoresis, NMR spectroscopy, and vibrational spectroscopy methods like IR, NIR, MIR, FTIR, and Raman [89].

The problem that occurs when the analysis of products for cutaneous application is concerned is the presence of an oil phase in almost all topical products, which makes sample preparation complicated. Liquid–liquid extraction is carried out using hexane, acetonitrile, and isopropyl alcohol as solvents [84,90]. Methanol as a polar solvent can be used for sample preparation without further purification via solid phase extraction in order to analyze the cannabinoid content in lotions, creams, shampoos, shower gels, lipstick products, essential oils, and hair colorants via the LC–MS technique [90].

GC–MS analysis is commonly used for the detection of the presence of cannabinoids and for their quantification [91]. Additionally, HPLCA-PDA, HPLC-fluorescence, and LC–MS can be used [84,90,92,93].

The labeling of CBD content in topical products typically indicates the total content within the entire product rather than the intended dose for each application. In a study conducted by Spindle et al., the THC content of 105 commercially available products was analyzed. It was discovered that in some of these products, the total THC amount reached up to 100 mg, which represents an intoxicating dose if ingested orally or inhaled. However, these results pertain to the entire product, and it is improbable that an individual would use the entire amount of the product in a single instance. Nevertheless, this raises concerns about potential misuse [91].

Several studies have found discrepancies between the claims made on cutaneous application products and their actual composition, reflecting a substantial issue amidst the abundance of such products in the market [93]. A surveillance study in Taiwan, which included 90 cosmetic products with hemp seed oil or hemp seed extract, revealed that THC was detected in 22 samples, out of which 50% had THC concentrations over 0.1%. Also, 6 out of 9 products with labeled CBD content had a CBD concentration within 10% of the one claimed [85]. In research conducted in 2022 [91], a total of 105 products for cutaneous application present on the market (lotions, creams, balms, salves, and patches) were tested for CBD and THC content in order to evaluate their label accuracy and quantify the stated claims. The results have shown that most of the products were inaccurately labeled when it comes to CBD content—18% of them had less than 10% of CBD than labeled, while 58% had more than 10% of labeled CBD. Some products were labeled as “THC-free”, or THC was not listed in the ingredients list at all. Regardless, THC was found in 35% of the analyzed products. However, in all of them, the THC concentration was under 0.3%, which represents the legal limit. While 14% of the products made cosmetic claims about wrinkle correction and skin nourishment, twice as many products (28%) made therapeutic claims, mostly about anti-inflammatory and analgesic activity. So far, the FDA has not authorized pain as an indication for using topical cannabinoid-based products; therefore, analgesic claims on them were not legally approved [91]. In a study conducted in Canada, two out of 40 tested cosmetic products had THC levels above the legal limit of 0.1%. CBD concentration varied from 0.19 to 8410 μg/g, suggesting that standard hemp testing should encompass both CBD and THC for prudence. Furthermore, assessing the ratios of CBD:CBDA and THC:THCA could serve as a useful diagnostic measure for potential adulteration [84].

## 8. Key Challenges and Advancements in the Development and Manufacturing Process

Numerous hurdles arise in the development and manufacturing processes of hemp-based topicals. To begin with, regulatory frameworks, which are not only highly diverse but also inconsistent globally and often reliant on specific country regulations, pose significant challenges for manufacturers aiming to create products suitable for the international market [82]. Another complication is achieving the desired cannabinoid content in these products. Variations in plant genetics and extraction methods, coupled with the inherent instability of cannabinoids and the presence of impurities, make it difficult to maintain consistent CBD levels. Cannabinoids are susceptible to degradation and oxidation, primarily induced by exposure to light or air. The choice of an appropriate extraction method can profoundly impact the chemical composition of the resulting extract [94].

Moreover, since it was shown that many ingredients can influence the cannabinoid absorption rate, it is important to further explore the influence of skin product formulation on cannabinoid absorption and the potential side effects of unwanted transdermal delivery [91]. Research needs to be conducted on whether single or repeated topical applications of products containing THC can lead to intoxicating serum levels. 

In addition, the question remains whether the application of topical products with CBD can lead to positive drug tests for cannabis. It was found that taking oral CBD products based on hemp, even with a low amount of THC (0.02%), can cause positive results on urinary drug tests due to the detection of THC metabolites [95]. This is in accordance with the findings about the CBD plasma levels measured after topical gel application [96].

## 9. Health Risks Associated with Cutaneous Application of Hemp

The development of a regulatory framework that focuses on the cutaneous application of hemp in terms of topical formulations in medicine and cosmetics is under way and still to be developed [97]. Some of the major concerns are related to improper labeling and a lack of scientific findings on the long-term use effects of hemp-based products in dermathopharmaceutical products, causing uncertainties regarding their safety and bioavailability [98,99]. Besides existing, promising positive confirmation reports on topical products that contain hemp-based or synthetic cannabinoids for the treatment of skin-associated diseases, optimization of formulations, dosages, and treatment instructions is still under investigation [57,96,100]. Moreover, possible adverse effects on health and systemic entry into circulation of these products are yet to be proved [34].

Spindle et al. [91] evaluated 60 online and 45 retail store-available hemp-derived topical products based on their cannabinoid content and labeling accuracy. Their results showed that only 21% showed the actual amount of CBD, whereas 18% showed less and 58% more content than what was on the label. In 37 examined products, out of 105, THC was detected. The presence of tetrahydrocannabinol in skin care products should be present only at trace levels due to its psychoactive compound nature [101]. Regarding CBD content, there are reports from Ali et al. [57] and Cohen et al. [102] confirming that CBD doses applied to patients with acne vulgaris had no observed adverse health effects.

In determining the cutaneous application of hemp’s possible adverse effects, it is essential that, besides CBD and THC content, quality control of used products consider the determination of possible contaminants such as pesticides and heavy metal residues or the presence of bacterial and fungal contamination [103]. Moreover, the risks of product allergenicity and CBD metabolite interaction with enzymes synthesized by the liver should be evaluated.

## 10. Future of Hemp Products for Cutaneous Application within Circular Bioeconomy Context

The pharmaceutical and cosmetic sectors are increasingly acknowledging the detrimental effects of excessive exploitation of natural resources for raw materials on the environment. This realization has prompted a quest for sustainable alternatives rooted in the principles of the circular bioeconomy [104,105].

Hemp, renowned for its versatility, stands out as an environmentally sustainable crop and a potent biomass converter capable of absorbing up to 22 tons of CO_2_ per hectare [106]. Hemp plant raw material has the potential to serve as a valuable source of active compounds for formulating both medicinal and cosmetic topical products. A 2019 report by the Brightfield Group stated that sales of CBD topicals in the United States exceeded USD 703 million, positioning them as the second-best-selling category of CBD products after tinctures [107]. As research into hemp extracts, hemp seed oil, and isolated hemp actives continues to expand, there will be a clearer understanding of their chemical composition and mechanisms of action. This enhanced understanding will facilitate the development of diverse and more potent hemp-based topicals. Anticipated advancements in delivery systems and nanotechnology suggest improved penetration of phytocannabinoids, potentially broadening the scope of indications for hemp-based products [9,44]. Projections suggest that in the United States, by 2025, the hemp-based products for cutaneous application market will increase to USD 4.5 billion [107].

Within the realm of manufacturing hemp-derived topicals, integrating the circular bioeconomy framework holds promise for enhancing resource efficiency, minimizing waste, and establishing a closed-loop system [108] wherein hemp production by-products are repurposed. This approach fosters a more sustainable and eco-conscious manufacturing process. The circularity principle extends beyond simply extracting NBCs for topicals; it encompasses utilizing various components of the hemp plant, such as fibers for packaging, seeds for oil-based formulations, and crop waste for compost or bioenergy generation [109,110]. Embracing this circular approach positions the manufacturing of hemp-derived topicals as a leading example of integrating circular bioeconomy principles into the emerging field of natural bio-compound-based products. This strategy ensures sustainability across all stages of production, addressing environmental, economic, and social concerns holistically. Local communities stand to benefit from the cultivation and processing of hemp crops, offering economic opportunities and fostering regional growth [111]. Furthermore, prioritizing sustainable practices nurtures consumer awareness and preference for environmentally friendly products [112,113], thereby boosting market demand for circular and bio-based solutions. By aligning hemp-based topical manufacturing with circular bioeconomy principles, the industry can spearhead a resilient and regenerative future, harmonizing economic prosperity, ecological vitality, and societal well-being.

Despite the promising outlook, the development and manufacturing of hemp products for cutaneous application within a circular bioeconomy face numerous challenges. Complex regulations, uncertain markets, and restricted supply chains hinder industry expansion and adaptability. Addressing these obstacles demands cooperation among stakeholders, such as policymakers, researchers, and industry members, to simplify regulations, fund research and development, and create resilient supply chains [113]. Looking ahead, advancements in biotechnology, nanotechnology, and sustainable packaging offer exciting opportunities to further optimize hemp-based formulations and enhance their environmental footprint [114].

## 11. Conclusions

The development and manufacturing of products for cutaneous applications containing hemp actives is an exciting area of research and innovation. Hemp, specifically its CBD content, has shown promise in providing therapeutic benefits for various conditions when applied topically. To create hemp-based products for cutaneous application, rigorous quality control measures must be implemented throughout the manufacturing process. This includes sourcing high-quality hemp extracts from reputable suppliers and ensuring that they meet strict purity and potency standards.

Formulation development plays a crucial role in creating effective products for cutaneous applications. Different delivery systems, such as creams, lotions, gels, or patches, can be explored to optimize the absorption and penetration of hemp actives into the skin. Additionally, the formulations may include other ingredients like emollients, humectants, and skin-conditioning agents to enhance the product’s texture, stability, and overall efficacy. Manufacturers must also consider stability testing and packaging requirements to ensure that the product maintains its quality throughout its shelf life. Adequate packaging, such as airless pumps or opaque containers, can protect the product from light and air exposure, which can degrade the hemp actives.

Regulatory compliance is of utmost importance when developing products for cutaneous application, where it is essential to adhere to local regulations and guidelines to ensure the safety, efficacy, and proper labeling of the products. When it comes to hemp-based skin products, the regulatory situation is even more complicated, while regulations are inconsistent globally and often country-specific.

Overall, the development and manufacturing of products for cutaneous application containing hemp actives require a meticulous approach to ensure quality, efficacy, and regulatory compliance. With further research and innovation, these products have the potential to provide safe and effective solutions for various dermatological conditions and improve overall skin health.

## Figures and Tables

**Table 1:** The main cannabinoids are present in the hemp extracts.

	Type of Cannabinoids	R1	R2	R3
Cannabigerol	Cannabigerolic acid (CBGA)	COOH	C5H11	H
	Cannabigerolic acid monomethyl ether (CBGAM)	COOH	C5H11	CH3
	Cannabigerol acid monomethyl ether (CBGM)	H	C5H11	H
	Cannabigerovarinic acid (CBGVA)	COOH	C3H7	H
	Cannabigerovarin (CBGV)	H	C3H7	H
Cannabichromene	Cannabichromene acid (CBCA)	COOH	C5H11	-
	Cannabichromene (CBC)	H	C5H11	-
	Cannabichromevarinic acid (CBCVA)	COOH	C3H7	-
	Cannabichromevarin (CBCV)	H	C3H7	-
Cannabidiol	Cannabidiolic acid (CBDA)	COOH	C5H11	H
	Cannabidiol (CBD)	H	C5H11	H
	Cannabidiol monomethylether (CBDM)	H	C5H11	CH3
	Cannabidiol-C4 (CBD-C4)	H	C4H9	H
	Cannabidivarinic acid (CBDVA)	COOH	C3H7	H
	Cannabidivarin (CBDV)	H	C3H7	H
	Cannabidiorcol (CBD-C1)	H	CH3	H
Delta-9-tetrahydrocannabinol	Delta-9-tetrahydrocannabinolic acid A (THCA-A)	COOH	C5H11	H
	Delta-9-tetrahydrocannabinolic acid B (THCA-B)	H	C5H11	COOH
	Delta-9-tetrahydrocannabinol (THC)	H	C5H11	H
	Delta-9-tetrahydrocannabinolic acid-C4 (THCA-C4)	COOH (H)	C4H9	H (COOH)
	Delta-9-tetrahydrocannabinol-C4 (THC-C4)	H	C4H9	H
	Delta-9-tetrahydrocannabivarinic acid (THCVA)	COOH	C3H7	H
	Delta-9-tetrahydrocannabivarin (THCV)	H	C3H7	H
	Delta-9-tetrahydrocannabiorcolic acid (THCA-C1)	COOH (H)	CH3	H (COOH)
	Delta-9-tetrahydrocannabiorcol (THC-C1)	H	CH3	H
Delta-8-tetrahydrocannabinol	Delta-8-tetrahydrocannabinol type of cannabinoids	-	-	-
	Delta-8-tetrahydrocannabinolic acid (Δ^8^-THCA)	COOH	C5H11	-
	Delta-8-tetrahydrocannabinol (Δ^8^-THC)	H	C5H11	-
Cannabinol and cannabinodiol	Cannabinolic acid (CBNA)	COOH	C5H11	
	Cannabinol (CBN)	H	C5H11	
	Cannabinol-C4 (CBN-C4)	H	C4H9	
	Cannabivarin (CBV)	H	C3H7	
	Cannabinol-C2 (CBN-C2)	H	C2H5	
	Cannabiorcol (CBN-C1)	H	CH3	
	Cannabinodiol (CBND)	-	-	C5H11
	Cannabinodivarin (CBVD)	-	-	C3H7

**Table 2:** Characteristics of CB1 and CB2 skin receptors [3,9].

	CB1	CB2
Location	Keratinocytes (stratum spinosum, stratum granulosum) Differentiated sebaceous cells Epithelial cells Hair follicular cells	Basal keratinocytes Undifferentiated sebaceous cells Undifferentiated infundibulum hair follicle cells
	Melanocytes Dermal fibroblasts Myoepithelial cells of eccrine sweat glands Sensory neurons Immune cells	
Function	Nociceptive activity Neural activity Anti-inflammatory activity by downregulation of cytokine production	Anti-inflammatory activity by inhibition of macrophage-1 polarization

**Table 3:** Biological activities of hemp derivatives on skin.

Compound	Activity	Mechanism	Type of Study	Ref.
CBD	antioxidant	Reduction in ROS formation in keratinocytes via NRF2-hemoxigenase-1 signaling pathway and proteasomal degradation of the transcriptional repressor BACH1, keratinocyte differentiation, skin development, epidermal cell differentiation, anti-proliferative activity	In vitro (primary human keratinocytes)	[11]
		Cytoprotective effect against UV-induced damage via activation of antioxidant response and signaling pathways	In vitro assay on 2D cultured human skin fibroblasts, CRL-1474 models	[12]
		Protection from lipid peroxidati	Epidermis cells from skin tissues were collected from 30 untreated patients with a diagnosis of psoriasis vulgaris and 15 healthy volunteers	[13]
	anti-inflammatory	Inhibition of (nuclear factor kappa B) NF-κB and genes that encode the expression of cytokines and metalloproteinases	In vitro assay on the immortalized murine BV-2 microglial cell line	[14]
		Inhibition of nuclear factor kappa B (NF-κB) and its inflammatory signaling pathway via stimulation of kinase IKK (IκB kinase)	In vitro (3D cultured human skin fibroblasts, CRL-1474 models)	[12]
	Wound healing	Simulation of keratinocyte proliferation by increasing the levels of hemoxigenase-1, keratins K16, and K17, resulting in an increase in skin thickness	In vivo animal study (mice)	[11]
	anti-age activity	Cytoprotective activity against UV-induced changes in fibroblasts, prevention of collagen degradation via activation of the PI3K/Akt pathway	In vitro (2D and 3D cultured human skin fibroblasts, CRL-1474 models)	[12]
	sebostatic activity	Sebostatic and anti-inflammatory effects on the pilosebaceous unit by activating both transient receptor potential vanilloid-4 (TRPV4) ion channels and adenosine A2a receptors on sebocytes	In vitro study on cultured human sebocytes	[32]
	UV photoprotection	Cytoprotection after the UVA irradiation of the cells	Primary cultures of human skin fibroblasts and the immortalized human keratinocyte cell line (HaCaT)	[10]
	moisturizing effect	Increase in expression of aquaporin-3, which contributes to skin water retention	In vivo (application for 14 days on 12 male HR-1 hairless mice)	[20]
	hair growth modulation	Concentration-dependent activity—submicromolar concentrations of CBD reduced hair loss via activation of adenosine receptor and reduction in intrafollicular cytokines production, while micromolar (≥10 μM) concentrations of CBD inhibited hair growth by activation of TRPV4	Ex vivo study on microdissected, organ-cultured human scalp and primary human outer root sheath keratinocytes isolated and cultured from plucked hair shafts	[17]
	antimicrobial activity	Active against Gram-positive bacteria	Antimicrobial activity bioassay	[25]
CBD and THC	wound healing	Promotion of wound healing	In vitro scratch assay on healthy and stress-induced premature senescent (SIPS) CCD-1064Sk skin fibroblasts and replicative senescent adult human dermal fibroblasts, CCD-1135Sk	[19]
	anti-senescence activity	Reduction in beta-galactosidase and cyclin D1 activity, cellular senescence biomarkers	In vitro assay on healthy and stress-induced premature senescent (SIPS) CCD-1064Sk skin fibroblasts with a β-Gal Detection Kit (Cat# AB176721, Abcam, Cambridge, UK)	[19]
	anti-age activity	Alteration of genes involved in extracellular matrix production, upregulation of genes involved in collagen, elastin, and hyaluronan production	In vitro assay on healthy and stress-induced premature senescent (SIPS) CCD-1064Sk skin fibroblasts exposed to CBD or THC	[19]
CBN	UV photoprotection	Cytoprotection after the UVB irradiation of the cells	Primary cultures of human skin fibroblasts and the immortalized human keratinocyte cell line (HaCaT)	[10]
CBD, CBN, CBG, CBC and THC	antioxidant	Antiradical activity and reducing potential—lipophilic electron-donors	Electrochemical measurement (square-wave voltammetry with the working electrode being a glassy carbon electrode)	[10]
THC, CBD, CBN, CBG	anti-proliferative activity	Inhibition of keratinocyte proliferation via PPARγ receptors and not by CB1/CB2 receptors	In vitro keratinocyte sulforhodamine B proliferation assay on HPV-16 E6/E7 transformed human skin keratinocytes	[33]
CBN, CBC, and CBG	skin whitening	Downregulation of tyrosinase activity	In vitro tyrosinase inhibitory activity assay with mushroom and murine tyrosinase	[21]
		Reduction in extracellular and intracellular melanin content via reduction in melanin synthesis	In vitro—murine melanoma B16F10 cells treated with α-melanocyte-stimulating hormone	[21]
CBN, CBC, CBG, and CBD	anti-melanoma activity	Cytotoxicity of CBN and CBC is comparable to the cytotoxicity of 5FU; cytotoxicity of CBG and CBD is twice the cytotoxicity of 5FU	In vitro cell viability assay (Neutral red uptake assay) of viability of human malignant melanoma A375 cells	[21]
CBDA from methanol hemp flower extracts	antioxidant	Radical scavenger towards DPPH^●^ free radicals	In vitro DPPH radical scavenging activity assay with HPTLC chromatogram	[22]
CBDA and cannabidivarinic acid (CBDVA) from methanol hemp flower extracts	skin whitening	Inhibition of tyrosinase activity	In vitro tyrosinase inhibitory activity assay (HPTLC chromatogram sprayed with L-DOPA substrate solution)	[22]
	antimicrobial activity	Strong activity against Gram-negative *Aliivibrio fischeri* bacteria and against Gram-positive *Bacillus subtilis*	*A. fischeri* bioassay combined with HPTLC, HPTLC-antimicrobial Bacillus subtilis bioassay	[22]
CBDA	antimicrobial activity	Active against *Bacillus subtilis*	Diffusion method with filter paper disks on nutrient agar medium	[27]
Hemp essential oil	antimicrobial activity	Strong activity against *Bacillus subtilis*, *Staphylococcus aureus*, and *Pseudomonas aeruginosa*, moderate activity against *Escherichia coli*	Cup-plate agar diffusion method	[29]
Hemp seed oil (ω-3, -6, -9 PUFA, CBD, β-caryophyllene, γ-tocopherol)	antimicrobial activity	Active against *Micrococcus luteus* and *Staphylococcus aureus* susp. *aureus*	Disc diffusion method and broth microdilution method	[30]
		Inhibition of the growth of the yeast *Saccharomyces cerevisiae*	Antimicrobial activity bioassay using yeast cells cultivated in medium	[31]
